# Physiological and geometrical effects in the upper airways with and without mandibular advance device for sleep apnea treatment

**DOI:** 10.1038/s41598-020-61467-4

**Published:** 2020-03-24

**Authors:** Adela Martínez, Alfonso López Muñiz, Eduardo Soudah, Juan Calvo, Alberto Álvarez Suárez, Juan Cobo, Teresa Cobo

**Affiliations:** 1 Departamento de Morfología y Biología Celular, Universidad de Oviedo, Asturias, Spain; 2 International Center for Numerical Methods in Engineering (CIMNE), Barcelona, Spain, C/Gran Capità, s/n, 08034 Barcelona, Spain; 3 Departamento de Resistencia de Materiales y Estructuras en la Ingeniería. Universidad Politécnica de Cataluña, C/Jordi Girona, 1-3, 08034 Barcelona, Spain; 4 Department of Radiology, Hospital Universitario Central de Asturias, Oviedo, Asturias, Spain; 5 Department of Mechanical Engineering. Escuela Politécnica Superior de Ingeniería. Universidad de Oviedo, Asturias, Spain; 6 Departamento de Cirugía y Especialidades Médico-Quirúrgica. Universidad de Oviedo, Oviedo, Spain; 7 Instituto Asturiano de Odontología, C/Julián Clavería 6, 33006 Oviedo, Spain

**Keywords:** Diagnosis, Biomedical engineering

## Abstract

Sleep apnea is a sleep disorder that occurs when the breathing of a person is interrupted during the sleep. This interruption occurs because of the patient has narrowed airways and the upper airways muscles relax, closes in and blocks the airway. Therefore, any forces or reaction originated by the air flow dynamics over the relaxed upper airways muscles could make to close the upper airways, and consequently the air could not flow into your lungs, provoking sleep apnea. Fully describing the dynamic behavior of the airflow in this area is a severe challenge for the physicians. In this paper we explore the dynamic behavior of airflow in the upper airways of 6 patients suffering obstructive sleep apnea with/without a mandibular advancement device using computational fluid dynamics. The development of flow unsteadiness from a laminar state at entry to the pharynx through to the turbulent character in the soft palate area is resolved using an accurate numerical model. Combining the airflow solution with a geometrical analysis of the upper airways reveals the positive effects of mandibular advance device in the air flow behavior (pressure drop). Improved modeling of airflow and positioning of mandibular advance device could be applied to improve diagnosis and treatment of obstructive sleep apnea.

## Introduction

Obstructive sleep apnea(OSA) is the most common form of sleep-disordered breathing disruption. OSA is characterized by repeated occurrences of upper airway(UA) collapse and obstruction during sleep^1^. It usually occurs when the muscles relax during sleep, causing soft tissue in the back of the throat to collapse and block the UA. With the objective to avoid apnea-episodes, the most common and popular treatment is Continuous Positive Air Pressure(CPAP). CPAP treatment splints the patient’s airway open during sleep by means of a flow of pressurized air into the UA. This solution forces the patient to wear a plastic facial mask connected by a flexible tube to a small bedside CPAP machine, however some patients find it extremely uncomfortable or do not tolerate it^2^. For this reason, nowadays orthodontists specializing in sleep disorders tend to prescribe a new patient-specific therapy based on a rapid maxillary expansion treatment using a Mandibular Advancement Device (MAD)^2,3^. And although the use of intraoral devices as MAD in the treatment of OSA is not a new concept, in 1902 Pierre Robin designed an oral device (the “monobloc”) in order to change the morphology of the UA with the objective to provoke a mandibular functional advancement to avoid the lingual fall backward (glossoptosis) and airway obstruction that appear during supine decubitus position^4^, during last years, several well controlled clinical trials have demonstrated its effectiveness^5–10^, These trials are hopeful, but at the same time, are difficult to standardize given the high variability of tolerance and individual response to treatment. Essentially, MAD is a custom-made mouthpiece that can shift the lower jaw forward/back allowing an opening up of the UA and nasal width, thus decreasing the resistance of nasal airflow. However, if the advancement of the jaw is excessive, the desired results may not be achieved. For that reason, understand the airflow dynamics inside the UA in MAD titration can play an important role in order to prevent non-desired outcomes. Considerable amount of work has been dedicated to understanding the airflow dynamics in the UA using computational techniques in real geometries^11–14^ or in phantom experimental and modelling^3,9,15^ for MAD titration. However, the exact changes in the pressure and velocities inside the UA during MAD treatment is yet to be fully understood. To understand better the airflow inside the UA when MAD is used, current work combines computed tomography images and computational fluid dynamics to compute the velocity and pressure along different cross-sectional views. To achieve this, 6 patients suffering mild-to-moderate apnea with and without MAD were analyzed. The objective is study the changes in the fluid dynamics inside the UA when a MAD is positioned. For each scenario the total volume, cross-sectional areas, tortuosity index, Reynolds number, stenosis ratio, velocities, pressure and flow resistance of the UA were studied. The computational model used in this study is based on a Steady Reynolds-Averaged Navier-Stokes (RANS) k-w model.

This paper is organized as follows. Section 2 presents the material and methods used for the simulation of OSA. In section 2 upper airway modelling, image reconstruction, meshing, and CFD simulation are presented. Next, in Section 3 the most significant results of the numerical simulation are summarized. Section 4 is devoted to summary and conclusions.

## Materials and Methods

### Participants and setting

**Figure 1:**
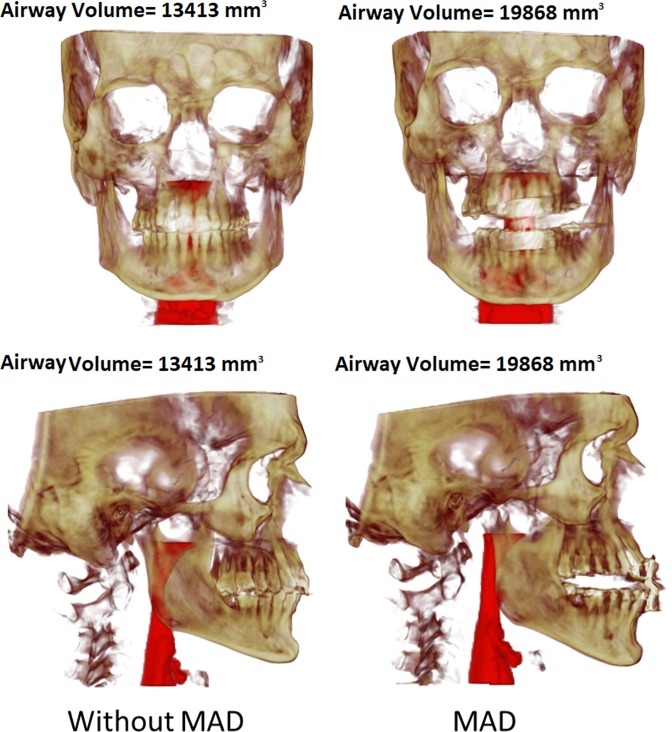
Front and lateral views of one patient. Left: without MAD; Right: with MAD (mandibular antepulsion).

A total of 6 patients with mild-to-moderate sleep apnea–hypopnea index (AHI) <30 h^-1^ were recruited to participate in this study. The average age of the 6 patients was 50 years ± 16 (means ± standard deviation), and their average body mass index (BMI ± kg/m^2^) was 26.1 ± 3.0. This research was reviewed and approved by the Ethics Committee of the Hospital Universitario Central de Asturias(HUCA) (Spain), and all the subjects provided written informed consent for this study. This manuscript has been prepared according to all ethical and scientific HUCA guidelines. For each patient, 2 CT medical images were acquired (Fig. 1): without MAD and with MAD in mandibular antepulsion, obtaining a total of 12 different scenarios.

### Upper airway modelling, ct scan and meshing

**Figure 2:**
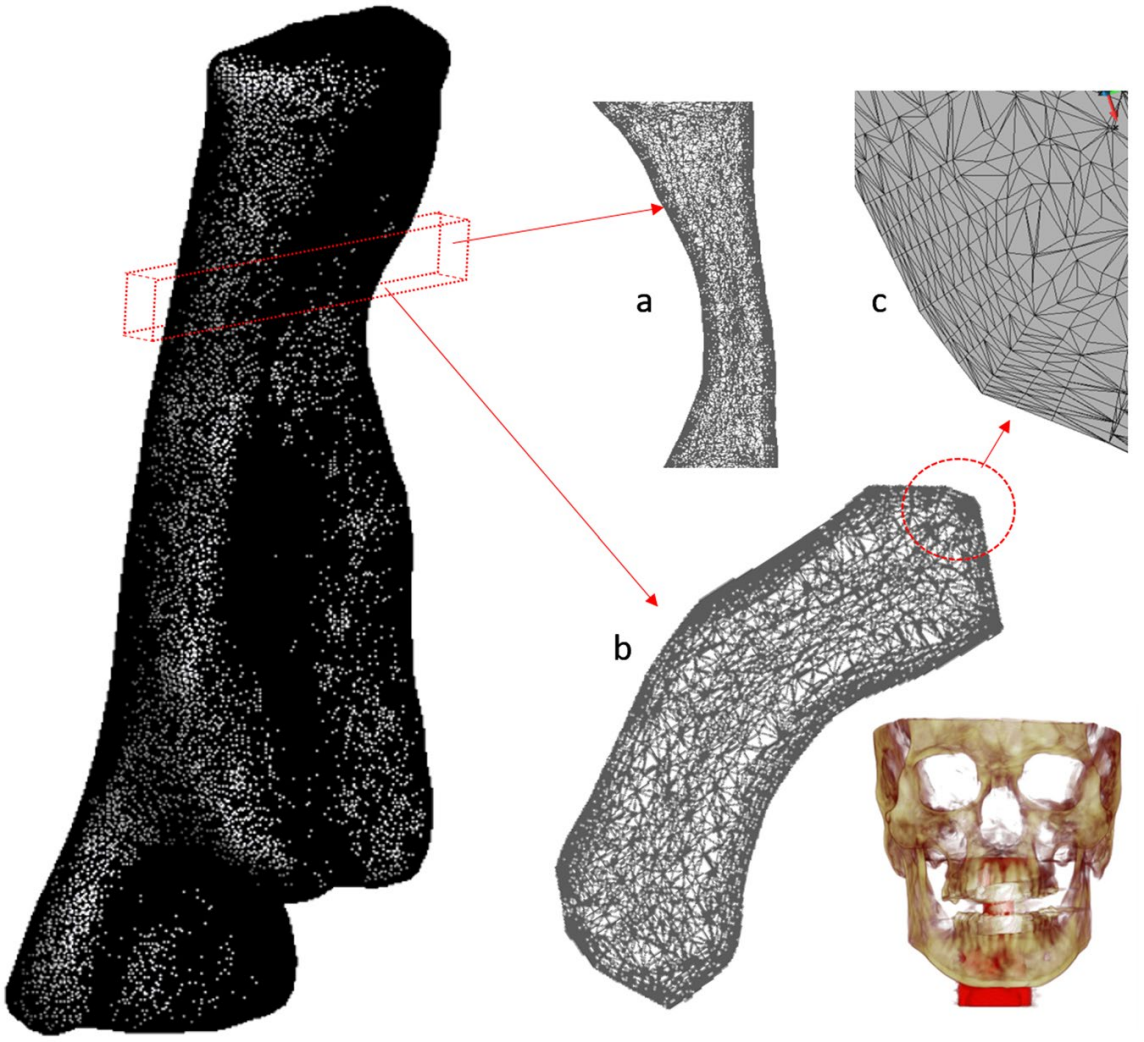
Sagittal view of upper airway mesh and details: (**a**) section in the sagittal plane above the pharynx, (**b**) detail of boundary layer, (**c**) section in the axial plane in the bottom part of the soft palate.

For the medical image acquisition, a Multidetector Helicoidal Computed Tomography (MDCT) Toshiba Aquilion 64 (Toshiba Medical Systems, Japan) was used with the following parameters: 512 × 512 × 491, pixel spacing: 0.468/0.468 with a resolution of 2.137 pixels per mm. Scanning was conducted, while the volunteer was awake, in the head first-supine position at the end of inspiration, using the protocol M.Facial-Parotid.tumoral. The scanning area covered all the UA, including the whole head. A special frame that holds the patient’s head in a fixed position have helped make the medical image more precise, and trying to keep fix the Frankfort plane before and after MAD positioning. The images were obtained one after the other, with and without MAD. The UA geometry was produced by semi-automatic segmentation of the DICOM images. The segmented area goes from the velopharynx (from the hard palate to the inferior tip of the soft palate) to the hypopharynx (from the epiglottis to the lowest portion of the airway at the larynx). Only the internal part of the pharynx was segmented. We left for a future paper the required improvements to simulate fluid-structure interaction problems. This surface was then smoothed using Taubin’s smoothing algorithm. Further details of the segmentation are provided by Soudah *et al*.^16^. The mesh generation software employed was GID^17^. All the meshes in this study were made of tetrahedrons elements with a boundary layer. A mesh sensibility analysis was performed to ensure the accuracy of the simulations, obtaining finally a 3D volume meshes consisting of 2.500.000 ± 10% tetrahedral elements depending on the complexity of the upper airway model. For the 12 acquisitions, the same medical image protocol, image processing and volume mesh technique were used. A detail of the computational mesh is shown in Fig. 2.

### Computational fluid dynamics

In the present work a Reynolds-Averaged Navier-Stokes (RANS) k-w model “tailored” specifically for meeting the peculiarities of the problem at hand is implemented in TDYN^18^. TDYN is based on a finite element model for the Navier-Stokes equations. The two equations k-w RANS model implemented into TDYN is based on the description done in Wilcox^19^. The two convective transport equations are solved for the turbulent kinetic energy and its specific dissipation rate, k and ω, respectively. And the turbulence length scale (L) is then determined as k^0.5^/ω. Usually, the appropriate value of k for an application is specified through a turbulence intensity level (TIL), which is defined by the ratio of the fluctuating component of the velocity (u’) to the mean velocity U. For low Reynolds number the turbulence intensity is typically between 1% and 5%. Due our conditions, we have used 1% as TIL. Our decision to choose the k-w model was driven by the fact that the k-ω model performs very well close to walls in boundary layer flows, particularly under strong adverse pressure gradients and to have a good performance in tubular structures as our models studied. Furthermore, and by the fact that the physiological parameters characterizing the mechanical behaviour (muscular tone) of the UA wall are not well determinate we decided to analyse only the worst situation (peak inspiration). Additionally, several studies have suggested different turbulences models to capture the pressure drop along the pharynx^11,16,20^. Air was modelled as incompressible, homogeneous and Newtonian. No external forces were applied (gravity was neglected). This approximation is acceptable as to mimic the density and viscous properties of air in UA. Air fluid characteristics were simulated with physiological parameters: density as 1.17 kg/m^3^ and viscosity as 1.8·10^-5^ kg/m·s (air flow at 25 °C). In the present work, we have computed a steady state simulation for the worst situation (peak inspiration).

### Boundary conditions

A velocity profile was settled at the upper part of the velopharynx(inlet) and a pressure at the bottom part of the hypopharynx(outlet). We consider that the oropharyngeal isthmus was closed, all the air flow is coming from the nasal cavities. For simulating the inlet profile, a flow input waveform alters in a sine wave (U = U_i_sin(2π·f·t)), (total time: 5 seconds (inspiration 2 seconds, expiration 3 seconds) and frequency(f) 12 insp./exp. per minute) which corresponds to transient respiration mode in a breathing period at the inlet of the upper airway was used. The inlet velocity **U** was calculated to obtain the same total tidal volume of 500 mL for the entire breathing cycle of inspiration/expiration for each patient and scenario. The velocity profile for each case dependent only on the geometric configuration of the pharynx. The values of mean velocity **U** in the inlet are in the range 1.2–2.3 m/s, aligned well with those reported in other computational studies^7,15,21^. In Table 1 is shown the velocity values used for each case analysed. A no-slip condition (pharynx rigid model wall) was imposed on the surface of the UA (the velocity on the wall was settled as zero).

**Table 1:** Main flow characteristic for the six patients analyzed with and without MAD. DH: hydraulic diameter, Re: Reynolds number, R: Resistance, Q: Flow, A _inlet_: Inlet Area, A _outlet_: Outlet Area.

	Inlet Velocity (m/s)	Inlet Mean Pressure(P) (Pa)	RE (A_inlet_)	RE (A_min_)	DH (A_inlet_) (mm)	DH (A_min_) (mm)	Perimeter (﻿A inlet) (mm)	Total R = P/Q (Pa/m^3^)
P1	1.52	98.91	1596.73	4468.98	16.16	6.75	63.75	25.26%
P1_MAD	1.19	18.83	1302.39	2723.93	16.84	8.73	77.89	4.79%
P2	2.02	22.16	1568.41	2976.22	11.95	8.35	57.08	5.66%
P2_MAD	1.38	21.23	1458.22	1531.08	16.32	9.41	70.05	5.40%
P3	1.66	21.54	1560.27	2155.88	14.46	8.10	65.40	5.49%
P3_MAD	1.29	16.45	1220.62	1434.97	14.51	8.83	83.69	4.19%
P4	1.51	28.32	1185.04	2491.93	12.10	9.84	67.29	7.21%
P4_MAD	1.32	27.29	1088.95	2539.65	12.73	10.07	70.35	6.94%
P5	2.30	8.72	2067.33	1894.61	13.83	10.25	55.76	2.21%
P5_MAD	1.30	3.95	1383.46	1428.56	16.37	13.46	75.79	1.01%
P6	1.73	4.49	1553.10	1768.13	13.85	11.48	65.80	1.14%
P6_MAD	1.42	1.82	1699.39	1409.43	18.37	14.00	60.11	0.46%

In the present work, we have considered pharynx wall as rigid. Other studies considered the pharynx wall as a flexible wall^22,23^, but remark that the physiological parameters characterizing the mechanical behaviour (muscular tone) of the UA wall for a patient with sleep-apnea are not well determinate. The outlet boundary condition was settled to 0 atm. of pressure at the laryngopharynx. To make consistent comparisons and with the objective to analyse the influence of the MAD, in our study same boundary conditions were considered in all cases.

## Results

In the present section, the UA simulation results using the present numerical model are discussed. The generalized Newton-Raphson method was used to solve the governing system of equations. The model relies on Backward Euler time integration scheme and linear velocity-linear pressure finite elements equipped with convective and pressure stabilization following the Finite Increment Calculus approach^24^. Bi-conjugate gradient solver was used for the solution of linear systems. For the two scenarios analysed (with and without MAD), the UA the geometrical factors and flow characteristics were studied and compared. Flow characteristics are obtained at any point of the fluid domain.

### Geometrical factor

To characterize the structure of the pharynx the following geometrical parameters were measured: pharyngeal length (LP), pharyngeal cross-sectional areas (inlet, outlet and minimum), the pharyngeal perimeter for the minimum and the inlet sections, the stenosis ratio (relation between the inlet area and the minimum area, Eq. 1), tortuosity index (ε) (Eq. 2) (relation between the pharyngeal length (LP) and the hypothetical pharyngeal straight length (${\rm{\tau }}$) and ${\rm{\gamma }}$ is ratio between the LP with and without MAD (Eq. 3).

1 $$ \% \,Stenosis=\frac{{A}_{inlet}-{A}_{minimun}}{{A}_{inlet}}$$

2 $${\rm{\varepsilon }}=\frac{{\rm{LP}}-{\rm{\tau }}}{{\rm{\tau }}}$$

3 $${\rm{\gamma }}=\frac{{{\rm{LP}}}_{withoutMAD}}{{{\rm{LP}}}_{withMAD}}$$

**Figure 3:**
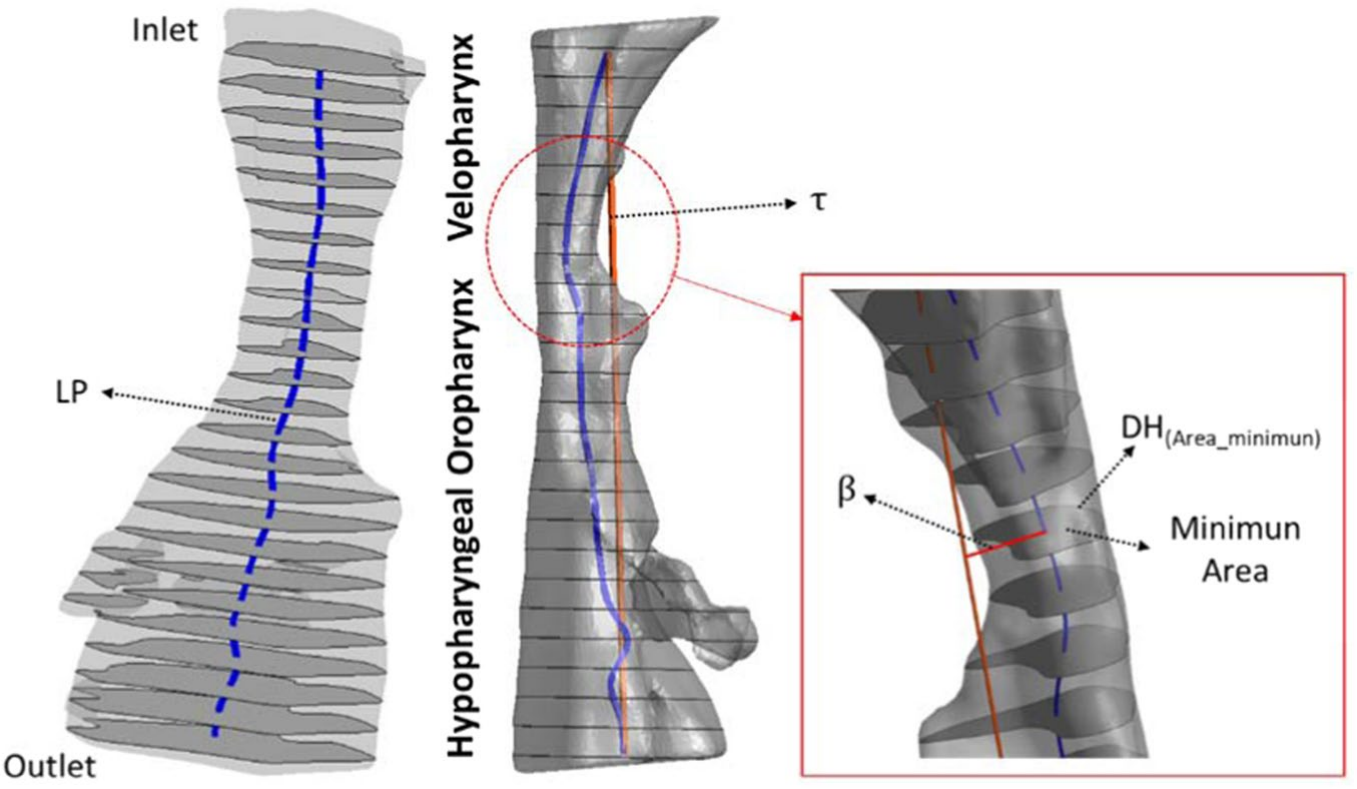
Schematic representation of the geometrical factor to characterize the pharynx.

To compute the centerline of the pharynx, we cut the pharynx in twenty-five cross-sectional areas (about 3 ± 0.25 mm each, depending on the case). The pharyngeal length (LP) is the length of the computed centreline. The hypothetical pharyngeal length pharynx (${\rm{\tau }}$) is the straight length between the first and the last center of mass from the twenty-five cross-sectional areas. The asymmetry factor (β), is the perpendicular distance of the center mass from the minimum area respect to the hypothetical pharyngeal straight line. Figure 3 shows a schematic representation of the ${\rm{\tau }}$ and LP length for patient 1. Geometrical factors are summarized in Table 2.

**Table 2:** Geometrical factors for the six patients analyzed (with and without MAD). LP: pharyngeal length, τ the hypothetical pharyngeal straight length, ε: tortuosity index, β:asymmetry factor, Amin: Minimum area, A inlet: Inlet Area, A outlet: Outlet Area and γ.

	Volume (mm^3^)	A_inlet_ (mm)	A_Outlet_ (mm)	A_min_ (mm)	τ (mm)	LP (mm)	Tortuosity (ε)	% Stenosis	β (mm)	γ
P1	11774.00	257.57	389.21	38.46	71.91	73.69	1.02	0.85	4.98	1.01
P1_MAD	21498.00	327.87	623.29	81.84	77.10	80.18	1.04	0.75	5.02	
P2	12043.00	193.90	380.91	71.39	68.47	73.76	1.08	0.63	3.42	0.96
P2_MAD	14397.00	285.73	199.25	156.87	67.14	69.66	1.04	0.45	2.65	
P3	13413.00	236.43	448.50	95.83	70.44	74.45	1.06	0.59	1.05	1.01
P3_MAD	19868.00	303.63	422.93	157.19	76.72	82.07	1.07	0.48	0.75	
P4	11937.60	203.48	430.28	100.83	66.76	71.52	1.07	0.45	0.36	0.98
P4_MAD	12046.00	223.85	412.65	101.29	63.50	66.92	1.05	0.55	0.40	
P5	15118.00	192.75	260.51	138.44	73.16	75.12	1.03	0.28	2.31	1.01
P5_MAD	30531.00	310.21	538.31	239.75	77.74	80.88	1.04	0.23	2.40	
P6	17883.00	227.81	350.78	165.82	71.80	72.75	1.01	0.27	0.93	1.00
P6_MAD	23698.00	276.00	405.37	253.81	80.27	81.28	1.01	0.08	1.60	

### Flow characteristics

Flow characteristics are summarized in Table 1. For all the cases (with and without MAD), the inlet velocity, mean pressure and the airway resistance(R) (Eq. 4) were calculated. The mean pressure(Eq. 6) was calculated for each sectional planes of the UA (total i-cross sectional planes, 25) (see Fig. 3). The airway resistance is the resistance of the respiratory tract to airflow during the inhalation and expiration, in our case, we use the peak flow inspiration, therefore we have compute the airway resistance just for the peak flow. Reynolds number, based on mean flow velocity and hydraulic diameter(DH) (Eq. 5), was calculated at the inlet and at the cross-section of the minimum area. The inlet velocity was calculated to obtain the same total tidal volume of 500 mL for the entire breathing cycle of inspiration/expiration for each patient.

4 $$R=\frac{\varDelta {P}_{total}}{Q}=\frac{{P}_{inlet}-{P}_{outlet}}{Q}$$

5 $$Re=\frac{DH\ast v}{\nu }$$

6 $${p}_{i}=\frac{{\iint }_{{{\rm{A}}}_{i}}{p}_{i}\,d{{\rm{A}}}_{i}}{{{\rm{A}}}_{i}}$$

The mean Reynolds numbers without MAD-with MAD at the inlet are (1588–1358) and, in the minimum cross-sectional area in the velopharynx are (2625–1844). These values corresponded to that observed in the literature^23,25,26^. Moreover, the values of mean maximum velocity magnitudes at the minimum cross-sectional area without and with MAD are (7.08–4.21) m/s, aligned well with those reported in other computational studies^23,26^.

**Figure 4:**
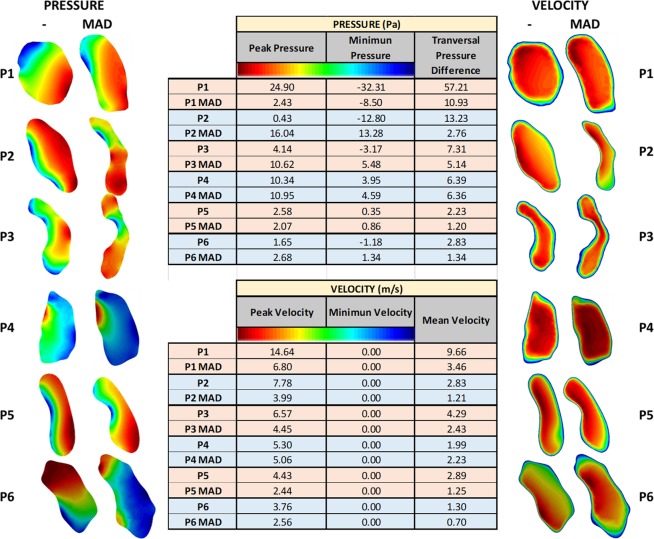
Cross-sectional area of the minimum area for the 6 patients (with and without MAD) analyzed.

Figure 4 shows the velocity and pressure (maximum, minimum and pressure difference) for the minimum cross-sectional area for each patient (with and without MAD).

**Figure 5:**
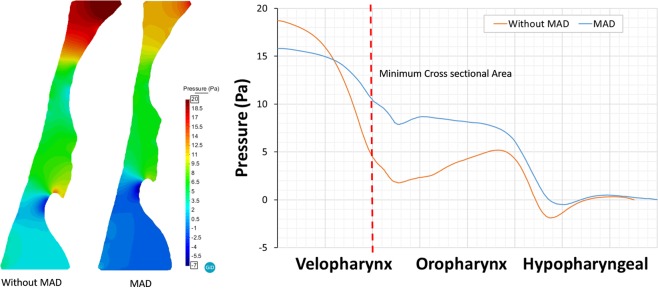
Computational fluid dynamics simulation result of patient 3 during peak inspiration (right, pressure gradient; left: pressure distribution along the centerline).

**Figure 6:**
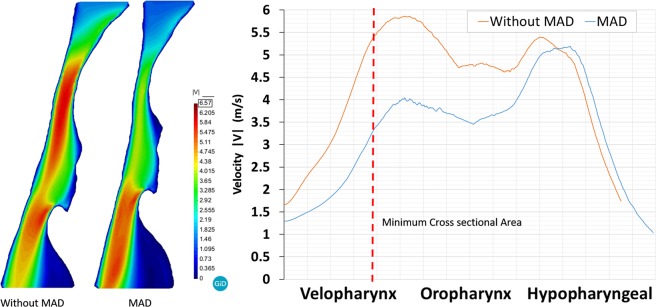
Computational fluid dynamics simulation result of patient 3 during peak inspiration (right, velocity distribution; left: velocity distribution along the centerline).

Figures 5 and 6 show the pressure and velocity distribution during the peak inspiration. Distal to the inlet, abrupt pressure drop and flow acceleration occur in the minimum area (velopharynx). Without MAD, the magnitude of the pressure drop from the inlet to the minimum area was larger than the pressure drop when MAD is used. Therefore, MAD decreases the pressure drop through the UA avoiding apnea episodes and improving breathing and oxygenation during sleep. Figure 5 shows the pressure distribution along the centerline in the mid-plane of the pharynx. Downstream of the A_min_ the pressure recovered gradually and fall dawn again in the epiglottis (top to hypopharyngeal zone).

Figure 6 shows the velocity distribution along the centerline in the mid-plane of the pharynx. When MAD is used we observe a de-accelerating of the air flow. The peak velocity reached without MAD is higher than when MAD is used. We observe that the device acts in the velopharynx area. Similar pressure and velocity behavior were observed for all the patients.

**Figure 7:**
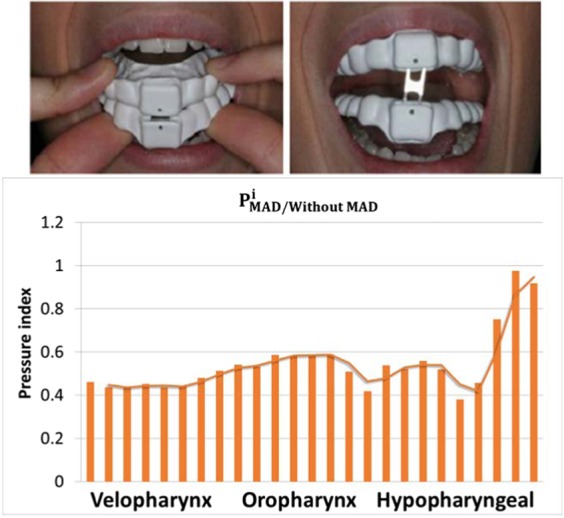
MAD Pressure index.

With the objective to check the MAD’s effectiveness (Fig. 7), a MAD pressure index was calculated for each i-cross sectional plane as:

$${P}_{MAD/Without\,MAD}^{i}=\mathop{\sum }\limits_{j=1}^{10}\frac{{P}_{j,{i}_{MAD}}}{{P}_{j,{i}_{WithoutMAD}}}\,\,\forall \,i=[1,25]\,\forall j=[1,6]$$

where ${P}_{j,{i}_{{Without\; MAD}}}$ and ${P}_{j,{i}_{{MAD}}}$ are the i-cross sectional mean pressure (Eq. 6) for the j-patient. *j* is number of the patient (total j-patient number, 6) and *i* is the number of the cross-sectional planes of the UA (total i-cross sectional planes, 25). Figure 7 shows the MAD device used for this study, and the pressure index ${P}_{MAD/Without\,MAD}^{i}$ for each cross-sectional plane. The values are normalized using the reference scenario (without MAD), therefore, values lower than one (<1) indicate that the pressure decrease improving the sleep-breathing (lower pressure drops). We observed that in the velopharynx area, the pressure drop decrease around 60%, and a constant gain rate around 50%. In the hypopharyngeal area, MAD pressure index is close to 1 because is our outlet boundary condition.

## Discussions

This study helps us investigate the influence of an oral appliance like MAD in the treatment of apnea combining functional medical images and computational models. In this work the computational model applied to the simulation of fluid flow in upper airway was a Reynolds-Averaged Navier-Stokes (RANS) k-w model. Two different scenarios were analyzed, with MAD and without MAD. The computational model allowed predicting the flow data (pressure and velocity) for the scenarios analyzed (see Table 1), and the image processing techniques allowed obtaining the pharynx geometrical factors (see Table 2). It is well known that higher airway resistance is usually associated with respiratory diseases, as sleep apnea or upper airway resistance syndrome^27^. Therefore, to decrease the airway resistance will imply less collapsibility, better breathing. From the cases analyzed, the use of the MAD increases the total pharynx volume, enlarged the minimum area (around the soft palate area) and provoke airflow changes in the UA. Related to the pressure, high pressure drop into the UA can provoke its collapsibility^1^. For all the patients (without MAD), high-pressure drop and flow acceleration goes up in the soft palate area. However, with MAD, pressure drop and velocity decrease. Therefore, MAD decreases the pressure drop through the UA avoiding apnea episodes and improving breathing and oxygenation during sleep. Based on the Reynolds number, we notice that when MAD is used the Reynolds number decreases around 30% in the minimum cross-sectional area, and a-priori the airway resistance of the upper airflow in the pharynx should be decreased. Such conclusion is however questionable since the geometry is varying (with and without MAD) and the range of the Reynolds number is close to the transitional regimen. Patients without MAD have Reynolds number higher than patients with MAD.

Detailed analysis of the airflow through the pharynx revealed that the jet at the soft palate area is typically shifted to the anterior part causing a decrease in the pressure in this region. This indicates that the pressure changes in the cross-sectional areas causing a transversal pressure differences (from the anterior to posterior areas) (see Fig. 4). This pressure difference between the anterior and posterior areas can cause the collapsibility of the pharynx. Remark that in this work, we have considered rigid wall and steady state during the peak inspiration. The mechanical properties of the pharynx wall are difficult to determinate, and therefore the compliance effects have not been considered for this study. This behavior is an important aspect that must be taken in consideration in the future numerical studies. Analysis of stress distributions in the area that is affected by jet emerging through the soft palate may provide valuable data for the clinical practice in terms of prediction of collapsibility in the external part of the upper airways. For patient 3, the total pressure drop without MAD is around 18 Pa, and with MAD is around 8 Pa. We observed that a negative pressure behind the epiglottis goes when MAD is not used (Fig. 5). This is as consequence that we are running an incompressible fluid, and we have set the operating pressure in 0 Pa at the bottom part of the hypopharyngeal area.

With the objective to evaluate the MAD’s effectiveness, a MAD pressure index was calculated. This index shows that the MAD used has a good performance to treat patients with mild-to-moderate sleep apnea. One of the main advantages of this device is that it allows different positions depending on the pathology of the patient. Although we found a strong correlation between geometry and the UA flow behavior, no clear conclusions of the asymmetry and tortuosity indices could be obtained. The main simplification of this work is that the pharynx wall is considered a non-deformable body. This simplification was taken as a result of unknowing the mechanical properties of the pharynx wall of the patients. Also for this study, we have not considered the nasal cavity due to the slight impact that the MAD has on it^12^. In a future work, we will consider the pharynx wall as a deformable body in order to simulate how it is collapsed and different MAD positions will be studied.

## Conclusions

This work gives a proof of the effectiveness of mandibular advancement device. A good correlation between the volume (areas), pressure drop and total pressure resistance when MAD is used is confirmed. The CFD airflow behavior in the UA obtained is similar to the airflow behavior observed when a more aggressive treatment (e.g. surgery) is applied. The proposed methodology has proven to be a feasible option for the analysis of the problem at hand and it can be used estimated the airway resistance instead of a body plethysmography.
